# Emergency open drainage of massive hemoperitoneum and early stage left hepatectomy for abdominal compartment syndrome due to hepatocellular carcinoma rupture: a case report

**DOI:** 10.1186/s40792-022-01478-7

**Published:** 2022-06-22

**Authors:** Makoto Kurimoto, Kenya Yamanaka, Masaaki Hirata, Makoto Umeda, Tokuyuki Yamashita, Hikaru Aoki, Yusuke Hanabata, Akina Shinkura, Jun Tamura

**Affiliations:** 1 Department of Surgery, Hyogo Prefectural Amagasaki General Medical Center, 2-17-77, Higashinaniwa, Amagasaki, Hyogo Japan; 2 Department of Gastroenterology, Hyogo Prefectural Amagasaki General Medical Center, 2-17-77, Higashinaniwa, Amagasaki, Hyogo Japan

**Keywords:** Spontaneous rupture, Hepatocellular carcinoma, Transcatheter arterial embolization, Abdominal compartment syndrome, Staged hepatectomy

## Abstract

**Background:**

Spontaneous rupture is one of the most life-threatening complications of hepatocellular carcinoma (HCC). Transcatheter arterial embolization (TAE) effectively achieves hemostasis in patients with hemodynamic instability. However, there have been no reports of abdominal compartment syndrome (ACS) caused by massive intra-abdominal hematoma after TAE. We report emergency open drainage of a massive hematoma for abdominal decompression and early stage left hepatectomy at the same time.

**Case presentation:**

A 75-year-old woman was transported to our emergency department with hypovolemic shock. Dynamic contrast-enhanced computed tomography revealed extravasation of contrast medium from a HCC lesion in the medial segment of the liver and a large amount of high-density ascites. TAE was immediately performed to achieve hemostasis. Three hours after the first TAE, we decided to perform a second TAE for recurrent bleeding. After the second TAE, the patient’s intra-abdominal pressure increased to 35 mmHg, her blood pressure gradually decreased, and she had anuria. Thus, she was diagnosed with ACS due to spontaneous HCC rupture. Twenty-seven hours after her arrival to the hospital, we decided to perform open drainage of the massive hematoma and left hepatectomy for ACS relief, prevention of re-bleeding, tumor resection, and intraperitoneal lavage. The operative duration was 225 min, and the blood loss volume was 4626 g. Postoperative complications included pleural effusion and grade B liver failure. She was discharged on postoperative day 33. The patient survived for more than 3 years without functional deterioration.

**Conclusions:**

Even after hemostasis is achieved by TAE for hemorrhagic shock due to spontaneous rupture of HCC, massive hemoperitoneum may lead to ACS, particularly in cases of re-bleeding. Considering the subsequent possibility of ACS and the recurrence of bleeding, early stage hepatectomy and removal of intra-abdominal hematoma after hemodynamic stabilization could be a treatment option for HCC rupture.

## Background

Spontaneous rupture of hepatocellular carcinoma (HCC) is thought to be caused by rapid tumor growth with necrosis, hepatic vein occlusion due to tumor thrombosis or vascular infiltration, or coagulopathy [1, 2]. Spontaneous rupture is a rare event that occurs in 2–3% of patients with HCC [3–5]. Despite recent improvements in management, mortality rates after bleeding events remain in the range of 16–30% [3, 5].

Many intra-abdominal bleeding events from HCC rupture can lead to hemodynamic instability and hemorrhagic shock. Hemodynamics is one of the factors that affect prognosis. The critical therapy is sufficient fluid resuscitation and control of tumor bleeding by transcatheter arterial embolization (TAE). TAE has been reported to effectively induce hemostasis in patients with hemodynamic instability, with a success rate of more than 90% [6]. TAE is considered a bridge to curative surgery in selected patients [7, 8]. However, the re-bleeding rate after primary interventional hemostasis including TAE is 22%, and the in-hospital mortality rate of the re-bleeding cases is 52% [5].

Organ dysfunction caused by intra-abdominal pressure (IAP) elevation is called abdominal compartment syndrome (ACS) [9]. Massive intra-abdominal bleeding due to HCC rupture increases IAP and may lead to ACS. However, there have been no reports of ACS caused by intra-abdominal hemorrhage due to spontaneous rupture of HCC after hemostasis by TAE treatment.

We describe a case of spontaneous rupture of HCC resulting in recurrent bleeding after the first TAE, and improved hemodynamics after the second TAE, with subsequent development of ACS. For the patient, we performed emergency open drainage of the massive hematoma for decompression and early stage left hepatectomy at the same time to prevent re-bleeding and improve the long-term prognosis.

## Case presentation

A 75-year-old woman with a chief complaint of epigastric pain from the previous night was transported to our emergency department early in the morning. Fluid resuscitation was initiated immediately, and noradrenaline was administered for hypovolemic shock. Her level of consciousness was assessed using the Japan Coma Scale I-3. After intubation, dynamic contrast-enhanced computed tomography (CT) was performed.

Eight months prior, she had undergone partial hepatectomy for HCC in segment 6 of the liver. The indocyanine green (ICG) retention test result was 14% (ICG-K = 0.137), and the Child–Pugh score was 5 (class A). Postoperative pathological findings showed no liver fibrosis (F0–1). Four months prior, transcatheter arterial chemoembolization was performed for recurrent HCC in segments 4 and 5. The HCC in segment 5 was fully controlled, but the HCC in segment 4 was increasing and was scheduled for retreatment. The Child–Pugh score was 5 (class A) after chemoembolization. The comorbidities included hyperlipidemia and bronchial asthma. She was not infected with hepatitis B or C viruses and had no history of habitual alcohol intake, nonalcoholic fatty liver disease, or autoimmune disease.

**Fig. 1:**
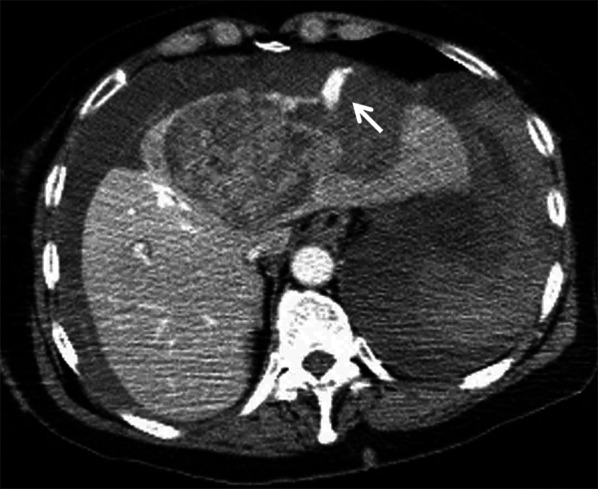
Findings of CT on arrival at the hospital. Dynamic contrast-enhanced CT scan showing extravasation of contrast medium from the ruptured tumor in the medial segment of the liver and a large amount of high-density intra-abdominal fluid collection

Enhanced CT revealed extravasation of the contrast medium from the known HCC lesion in the medial segment of the liver. A large amount of high-density ascites was observed in the abdominal cavity (Fig. 1). Thus, the patient was diagnosed with spontaneous rupture of HCC.

TAE was performed to control the bleeding from HCC rupture. No tumor staining was observed on examination of the right hepatic artery. The left inferior phrenic artery (IPA) and the left hepatic artery branching from the left gastric artery were embolized. Hemostasis was achieved and blood pressure was elevated; however, the IAP was 27 mmHg. Then, abdominal puncture was performed, resulting in the drainage of 3000 ml from the intra-abdominal hematoma; the IAP improved to 10 mmHg.

Three hours after the first TAE, the patient experienced hemorrhagic shock due to recurrent bleeding. A second TAE was performed. The left IPA regained flow, and rebleeding from the left IPA was observed. In addition, tumor staining from A8 was observed at this time. Then, the branches of A8 and the left IPA were embolized. The IAP after the second TAE procedure was 35 mmHg. Hemostasis was achieved, and hemorrhagic shock was fully controlled. However, her blood pressure tended to decrease, with anuria. Then, she was diagnosed with ACS.

Finally, 27 h after her arrival to the hospital, we decided to perform open drainage of the massive hematoma and left hepatectomy for ACS relief, prevention of re-bleeding, tumor resection, and intraperitoneal lavage. The preoperative hematological examination results are shown in Table 1.

The abdomen was opened by an inverted T-shaped incision using an incision under the right rib arch of the previous operation. We found and aspirated a large hematoma in the abdominal cavity. Bleeding from the ruptured tumor was not observed. The adhesions around segment 6 of the liver were detached. The hepatoduodenal ligament was identified and encircled for the Pringle maneuver. The main tumor consisted of a ruptured HCC lesion extending from the medial area to segment 3. No obvious HCC was palpable in the right lobe of the liver. No chronic hepatitis or cirrhosis was observed in the liver. The gallbladder was removed. The left hepatic artery was a replacement derived from the left gastric artery. The proper hepatic artery (PHA) was encircled, and the portal vein behind the PHA was identified. The left branch of the portal vein was encircled. We clamped the left branch of the portal vein and confirmed the demarcation line. The inferior vena cava (IVC) of the upper liver was dissected, and the root of the middle hepatic vein (MHV) and left hepatic vein (LHV) was identified. The left lobe of the liver was mobilized, and the replaced left hepatic artery was ligated and dissected. The left IPA was dissected with the hepatogastric ligament. We started the liver dissection and preserved the MHV. The V4 branch and the fissure vein branch were ligated and dissected. Since the venous pressure was high and some venous bleeding was observed, the infrahepatic IVC was exposed, and hepatectomy was performed under the clamp of the infrahepatic IVC. The caudate lobes were preserved, and the first branch of the left Glisson capsule was dissected using a Tri-Staple (Echelon FLEX™). The LHV was also dissected using a Tri-Staple (Echelon FLEX™), and the specimen was removed. We performed peritoneal lavage with saline. A fibrin sealant patch (TachoSil™) was attached to the cut surface of the liver. The operative duration was 225 min, and the blood loss volume was 4626 g. Blood transfusion with 6 units of packed fresh-frozen plasma (FFP) and 6 units of packed red blood cells (PRBCs) was performed.

**Fig. 2:**
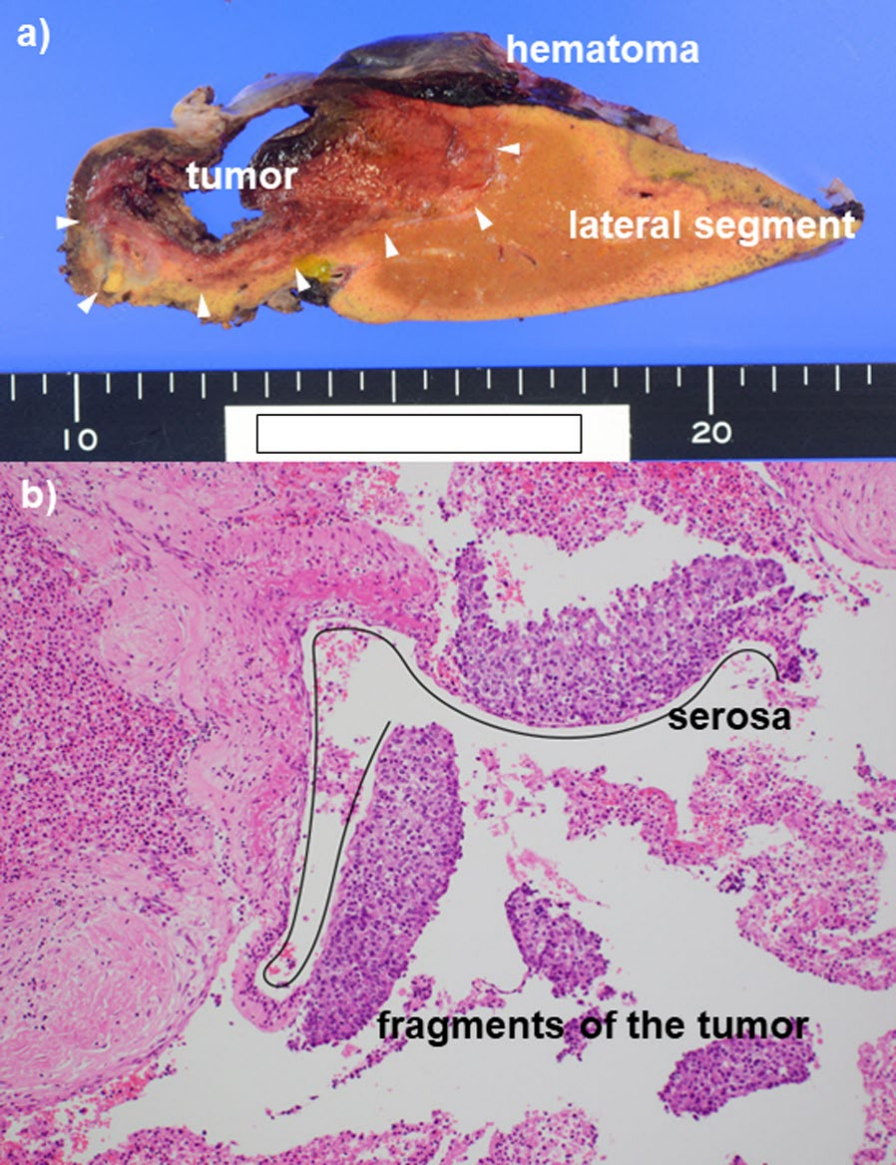
Postoperative findings as HCC rupture. **a** Macroscopic pathology. A subserosal hematoma on the tumor and the lateral segment was observed. The hematoma was attached to the necrotic tissue of the liver parenchyma. **b** Microscopic pathology. Moderately differentiated hepatocellular carcinoma (HCC) was observed. Since fragments of the tumors were found outside the broken serosa of the liver, HCC rupture was histopathologically diagnosed

The postoperative pathological diagnosis was HCC, moderately differentiated, and ruptured (Fig. 2a, b). The hepatic serosa was ruptured, and the hematoma was continuous with the liver parenchyma with necrotic tissue. No vascular infiltration was observed.

Regarding blood transfusion, 30 units of PRBCs, 30 units of FFP and 10 units of platelets were infused on the day of TAE in total, and 8 units of PRBCs, 12 units of FFP and 10 units of platelets were infused on the day of the surgery in total.

The postoperative complications included pleural effusion and grade B liver failure (the maximum serum total bilirubin level was 1.8 mg/dl) [10]. The patient was extubated 3 days after the surgery. She was discharged on postoperative day 33. Despite intrahepatic recurrence of HCC, 9 months after the surgery, the patient was treated with chemotherapy, which achieved a complete response. She survived for more than 40 months after spontaneous rupture of HCC without functional deterioration.

## Discussion

The treatment of spontaneous rupture of HCC has two missions: improvement of the mortality rate as a short-term treatment outcome and improvement of the prognosis as a long-term treatment outcome. In an emergency setting, lifesaving measures should be emphasized. However, to improve the long-term prognosis, it is necessary to consider treatment options for HCC after lifesaving, even in an emergency setting.

Hemodynamic instability, poor liver function, large tumor size, and no treatment with TAE have been reported to contribute to reduced 30-day mortality rates in patients with HCC rupture [11]. If liver function is impaired, bleeding may further exacerbate liver dysfunction, resulting in decompensated cirrhosis [2]. Bleeding and shock have a strong influence on patient prognosis. Thus, the first step is to control bleeding and shock to improve short-term treatment outcomes. Although conservative treatment may be recommended for patients who cannot undergo TAE, aggressive fluid resuscitation and TAE to protect the liver from further damage are essential for improving the prognosis [11]. In this case, initial treatment comprised fluid resuscitation and TAE. Because recurrent bleeding occurred after the first TAE, a second TAE was performed to control tumor bleeding.

Intra-abdominal hypertension is classified as follows: grade I, 12 to 15 mmHg; grade II, 15 to 20 mmHg; grade III, 20 to 25 mmHg; and grade IV, > 25 mmHg [9]. ACS is defined as a sustained IAP > 20 mmHg that is associated with new organ dysfunction/failure [9]. The transbladder technique is used as a standard IAP measurement technique [9]. Typical clinical symptoms of ACS include increased maximal airway pressure, oliguria, and decreased cardiac function [12]. The mechanism of progression from ACS to multiple organ dysfunction syndrome (MODS) is considered to be gastrointestinal damage due to increased IAP [13]. Dysfunction of the intestinal mucosal barrier causes the growth of intestinal bacteria, resulting in extensive organ dysfunction and tissue damage due to endotoxin [13, 14]. There have been several reports of MODS attributable to ACS after abdominal aortic aneurysm (AAA) rupture, and it is the main cause of death of endovascular aortic repair in patients with AAA rupture [12]. The massive transfusion of PRBCs has been reported as a risk factor for the development of ACS in cases of AAA rupture [15]. In our case, 38 units of PRBCs were transfused in total. Our case was classified as grade IV intra-abdominal hypertension. Decompression laparotomy is one of the standard treatments for ACS, because it quickly lowers the IAP and improves organ dysfunction [9]. The mortality rate is still high after rebleeding of HCC rupture [5], and it is necessary to consider the possibility that ACS is involved. Therefore, we decided to promptly remove the massive hematoma.

Staged partial hepatectomy was feasible in 12–26% of patients and had a 5-year overall survival rate of 15–33.9% [1, 8]. If liver function is preserved, staged hepatectomy is recommended by many surgeons for perioperative safety and acceptable long-term survival outcomes [16]. Regarding the evaluation of liver function, it has been reported that surgical treatment of HCC rupture patients with Child–Pugh class B disease should not be performed in consideration of the poor prognosis [6]. The evaluation of liver tumors depends on the characteristics and stages of each tumor [2, 11]. According to the guidelines for the management of HCC, hepatectomy is indicated in cases of up to three tumors, regardless of tumor size [17]. In our case, the stage and the characteristics of the tumor were included in the criteria for evaluating tumor resection. In addition, data on liver function before liver tumor rupture were available: liver function was found to be well maintained; ICG-K = 0.137, Child–Pugh class A, and no liver fibrosis. We consider ICG-K of remnant liver > 0.05 an indication for hepatectomy [18, 19]. Thus, we preoperatively evaluated whether postoperative liver failure after left hepatectomy could be avoided.

It has not been established when to perform staged hepatectomy after TAE. In Japan, staged hepatectomy is often performed after 1–3 months of follow-up to confirm the improvement in the hemoperitoneum and the absence of intrahepatic or peritoneal metastasis [6, 20]. On the other hand, some surgeons recommended that early stage hepatectomy should be performed within 7 days after TAE. Most bleeding recurrence events occur in the first week after initial TAE [5]. Considering the risk of re-bleeding and the associated high mortality, curative liver surgery should be discussed within a short time frame (7 days) after hemostasis [5]. In addition, patients who underwent staged partial hepatectomy after a delay of 8 days or more showed poorer long-term survival outcomes, and delayed hepatectomy was an independent risk factor for postoperative peritoneal dissemination [16]. Peritoneal lavage with saline can help reduce the likelihood of peritoneal spread of cancer, which may be a complication of TAE [2]. Early resection of a ruptured tumor and removal of an intra-abdominal hematoma may reduce the incidence of peritoneal dissemination [21]. In our case, a decompression laparotomy was required for the treatment of ACS. Moreover, we were concerned about the risk of bleeding recurrence and the risk of peritoneal dissemination if the tumor remained. Therefore, we decided to perform an early stage left hepatectomy at the same time as drainage of the massive hematoma.

Apart from staged hepatectomy, some surgeons recommend performing emergency hepatectomy if the patient's condition is applicable [6]. Emergency hepatectomy has been advocated to achieve both hemostasis and tumor removal. The rate of postoperative peritoneal dissemination has been reported to be higher in patients who underwent staged hepatectomy than in those who underwent emergency hepatectomy [16]. It has also been reported that in-hospital mortality from emergency hepatectomy has decreased in recent years due to improvements in diagnosis, perioperative care, and surgical techniques [16]. However, emergency hepatectomy without sufficient preoperative evaluation appears to be dangerous, as it can cause postoperative liver failure [22]. Thus, we believe that emergency surgery is not the first choice, because it is important to safely achieve hemodynamic stability due to intra-abdominal bleeding in consideration of the high success rate of hemostasis by TAE.

## Conclusions

We report a case in which hemostasis was achieved by TAE for hemorrhagic shock due to spontaneous rupture of HCC, which led to massive hemoperitoneum and the development of ACS. We performed emergency open drainage of the hematoma and early stage left hepatectomy. The patient survived for more than 3 years without functional deterioration. Considering the subsequent possibility of ACS after HCC rupture and the recurrence of bleeding, early stage hepatectomy and removal of intra-abdominal hematoma after hemodynamic stabilization could be a treatment option for HCC rupture.

**Table 1:** Preoperative hematological examination

Items	Results	Unit	Items	Results	Unit
WBC	3.96	×10^12^/L	AST	2440*	U/L
Hb	12.1	g/dL	ALT	1930*	U/L
PLT	82*	×10^9^/L	ALP	163	U/L
			LDH	2853*	U/L
PT	49.8	%			
APTT	73.6*	sec	Na	139	mmol/L
Fibrinogen	212	mg/dL	K	5.0*	mmol/L
			Cl	103	mmol/L
TP	5.3*	g/dL	Ca	8.7*	mg/dL
ALB	3.3*	g/dL	P	7.8*	mg/dL
T-BIL	1.5	mg/dL			
D-BIL	0.3	mg/dL	CRP	1.27*	mg/dL
AMY	479*	U/L	BNP	127.6*	pg/mL
CK	561*	U/L			
			pH -A	7.369	
BUN	25.1*	mg/dL	B.E. -A	− 3.6*	mmol/L
CRE	2.30*	mg/dL	Lac -A	4.1*	mmol/L
eGFR	17*	ml/min			

*WBC* white blood cell count, *Hb* hemoglobin concentration, *PLT* platelet count, *PT* prothrombin time, *APTT* activated partial thromboplastin time, *TP* total protein, *ALB* albumin, *T-BIL* total bilirubin, *D-BIL* direct bilirubin, *AMY* amylase, *CK* creatine kinase, *BUN* blood urea nitrogen, *CRE* creatinine, *eGFR* estimated glomerular filtration rate, *AST* aspartate aminotransferase, *ALT* alanine aminotransferase, *ALP* alkaline phosphatase, *LDH* lactate dehydrogenase, *CRP* C-reactive protein, *BNP* B type natriuretic peptide, *B.E.* base excess, *Lac* lactate

*Without normal range

## Data Availability

Not applicable.

## Abbreviations

AAA: Abdominal aortic aneurysm
ACS: Abdominal compartment syndrome
FFP: Fresh frozen plasma
HCC: Hepatocellular carcinoma
IAP: Intra-abdominal pressure
ICG: Indocyanine green
IPA: Inferior phrenic artery
IVC: Inferior vena cava
LHV: Left hepatic vein
MHV: Middle hepatic vein
MODS: Multiple organ dysfunction syndrome
PHA: Proper hepatic artery
PRBC: Packed red blood cell
TAE: Transcatheter arterial embolization
